# What happens to cardiovascular system behind the undetectable level of HIV viremia?

**DOI:** 10.1186/s12981-016-0105-z

**Published:** 2016-04-27

**Authors:** Gabriella d’Ettorre, Giancarlo Ceccarelli, Paolo Pavone, Pietro Vittozzi, Gabriella De Girolamo, Ivan Schietroma, Sara Serafino, Noemi Giustini, Vincenzo Vullo

**Affiliations:** Department of Public Health and Infectious Diseases, University of Rome “Sapienza”, Viale del Policlinico 155, Rome, Italy

**Keywords:** Cardiovascular diseases, cARV, HIV, Premature aging

## Abstract

Despite the combined antiretroviral therapy has improved the length and quality of life of HIV infected patients, the survival of these patients is always decreased compared with the general population. This is the consequence of non-infectious illnesses including cardio vascular diseases. In fact large studies have indicated an increased risk of coronary atherosclerotic disease, myocardial infarction even in HIV patients on cART. In HIV infected patients several factors may contribute to the pathogenesis of cardiovascular problems: life-style, metabolic parameters, genetic predisposition, viral factors, immune activation, chronic inflammation and side effects of antiretroviral therapy. The same factors may also contribute to complicate the clinical management of these patients. Therefore, treatment of these non-infectious illnesses in HIV infected population is an emerging challenge for physicians. The purpose of this review is to focus on the new insights in non AIDS-related cardiovascular diseases in patients with suppressed HIV viremia.

## A change of perspectives in the management of HIV infection: from an infectious disease to an internal medicine illness

The introduction of potent combined antiretroviral therapy (cART) has dramatically increased the survival of HIV infected patients and has changed their causes of morbidity and mortality from AIDS-related opportunistic infections to serious non AIDS events (SNAEs) [1].

Currently achieving undetectable viral load has become the minimum objective achievable with antiretroviral therapy; otherwise the safety of the therapy, the reduction of residual viremia, the eradication of the virus from the reservoirs and the reduction of the process of immune activation and chronic inflammation are still challenges for scientific research.

In particular to date is not well cleared the mechanisms for the establishment and maintenance of chronic immune activation. All available data suppose the presence of multiple and complex mechanisms acting synergistically and may contribute to the pathogenesis of SNAEs: viral factors, microbial translocation (MT), immune activation, systemic inflammation and side effects of therapy. Residual viremia, defined as the continuous persistence of the virus also at low levels despite effective cART, could be responsible of the chronic generalized inflammation and of the activation of the coagulation system. In addition the damage of mucosal barrier in the gastrointestinal tract (GI) increases MT into the circulating blood and increase the risk of systemic complication such as immune activation.

The deleterious effect of the status of immune activation on the host immune system is still unclear. Undoubtedly an important consequence of the general status of inflammation is the ability of HIV to infect and kill activated CD4 T-helper cells with the impaired homeostasis of CD4 T cells and memory B cells by the fibrosis of lymphoid tissue; all that may contribute to the consequent inability of the host to control a wide range of potential pathogens. In fact the persistent immune activation makes CD4 T cells more susceptible to infection and causes a vicious cycle increasing the production of soluble inflammatory markers such as IFN-γ, inflammatory cytokines (i.e. IL-6) and indoleamine 2,3-dioxygenase (IDO) [2–7].

**Fig. 1:**
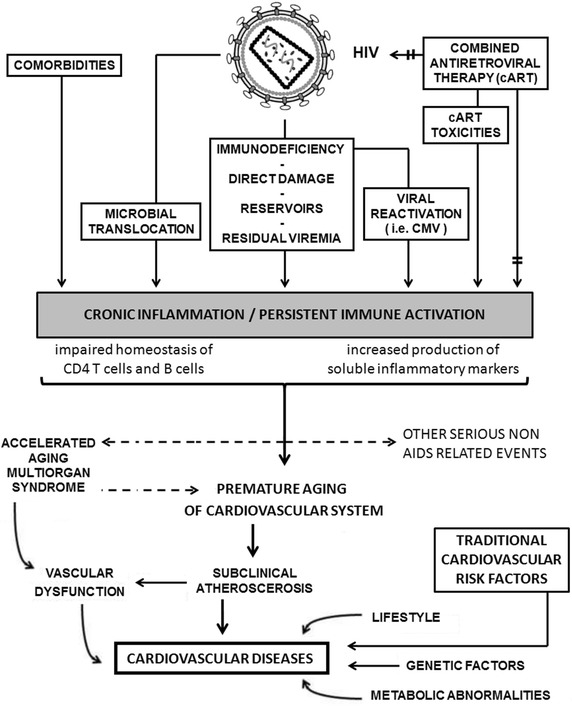
Hypothesis of premature and accelerated of cardiovascular system aging in cARV treated HIV-infected patients

The excellent results now routinely achieved by cART have made the occurrence of opportunistic infections increasingly less frequent; at this time however it is common to observe cases of patients with undetectable blood levels of HIV-RNA, significant recovery of immunological status and SNAEs including cardiovascular diseases (Fig. 1). For this reasons it becomes always more intriguing to know the pathogenesis and clinical characteristics of SNAEs during HIV infection to cure them.

## The cardiovascular disease: a challenge for the cure of HIV patients

### Epidemiology of cardiovascular diseases in HIV positive patients

A large body of evidences in scientific literature supports the epidemiological observation of an increase of morbidity and mortality related to clinical and sub-clinical cardiovascular diseases (CVD) in HIV patients in the era of cART. A significantly higher risk of acute myocardial infarction (AMI) was observed in HIV infected patients compared with uninfected in veterans aging cohort study virtual cohort (VACS-VC) that analyzed data collected in 82,459 veterans followed for an average of 5.9 years; unexpectedly the highest risk was found in patients in cART and with HIV-RNA less than 500 copies/ml [8]. Also Silverberg et al. [9] reported an higher risk of AMI among HIV+ patients with a low recent or nadir CD4 cells (<200) compared with HIV-subject in a cohort study population included 22,081 HIV positive and 230,069 HIV negative subjects.

In contrast Klein et al. [10] reported a decline of CVD in HIV patients as observed by the analysis of the data of Kaiser Permanente Southern California and Northern California health plans including 24,768 HIV positive patients and 257,600 controls. By these data the adjusted relative risk of AMI in HIV+ patients declined from 1.8 in 1996–1999 to 1.0 in 2010–2011 possibly due to the better control of the traditional CVD risk factors, the lower incidence of severe immunodeficiency, and the use of more lipid-friendly cART in the patients enrolled in this study.

Although the data reported by Klein et al. probably influenced by the specific characteristics of their cohort, epidemiological studies show a significant overall impact of HIV on CVD. Since the size of the problem (although decreased after the introduction of lipid free antiretroviral drugs) remains very important, the actual aim of HIV medical practitioners is to better understand the kind of cardiovascular (CV) prevention to be applied to HIV population.

Recent retrospective and prospective studies evaluating the epidemiology of CVD in HIV positive patients were reported in Table 1.

**Table 1:** Recent retrospective and prospective studies evaluating the epidemiology of cardiovascular diseases in HIV positive patients

Author, year [Ref.]	Cohort	Type of study	Population	Time of follow up	Aims	Results
Tseng et al. [96]	HIV+ patients enrolled in a public HIV Clinic in San Francisco in 10 years	Retrospective	2860 HIV+	3.7 years	To determine the incidence of SCD in HIV+ patients	Of 230 deaths, 13 % met SCD criteria
						SCDs accounted for 86 % of all cardiac deaths (30 of 35)
						Mean SCD rate: 2.6 per 1000 person-years (95 % CI 1.8–3.8), 4.5-fold higher than expected
Esser et al. [97]	HIV+ outpatients (ClinicalTrials.gov NCT01119729)	Prospective observational	803 HIV+	N/A	To elucidate CVD prevalence in HIV+ outpatients by standardized non-invasive CV screening	Prevalence of CVD: 10.1 % (95 % CI 8.0–12.2 %)
						Aging HIV-infected patients (≥45 years) exhibited significantly increased rates of CVD,
						CAD (7.5 vs. 1.8 %, p < 0.001)
						MI (6.0 vs. 1.8 %, p = 0.002)
						PAD (4.6 vs. 1.5 %, p < 0.017)
						Significantly associated with the prevalence of CVD in multivariate analyses:
						Age (OR 2.05 xd, 95 % CI 1.64–2.56)
						Smoking (OR 5.96 xd, 95 % CI 2.31–15.38)
						Advanced symptomatic HIV infection (OR 2.60 xd, 95 % CI 1.31–5.15)
Freiberg et al. [8]	Veterans aging cohort study virtual cohort (VACS-VC)	Prospective observational	55,109 HIV+ 27,350 HIV−	5.9 years	To investigate whether HIV is associated with an increased risk of MI	The mean MI events per 1000 person-years significantly higher (p < 0.05 for all) for HIV-positive compared with uninfected veterans:
						Age 40–49 years, 2.0 (1.6–2.4) vs. 1.5 (1.3–1.7)
						Age 50–59 years, 3.9 (3.3–4.5) vs. 2.2 (1.9–2.5)
						Age 60–69 years, 5.0 (3.8–6.7) vs. 3.3 (2.6–4.2)
						After adjusting for Framingham risk factors, comorbidities, and substance use, HIV-positive veterans had an increased risk of inc ident MI compared with uninfected veterans (hazard ratio, 1.48; 95 % CI, 1.27–1.72)
Silverberg et al. [9]	Kaiser Permanente California	Retrospective	22,081 HIV+ 230,069 HIV−	13 years	To evaluate association of HIV infection and immunedeficiency on MI risk	MI incidence rate per 100,000 person-years: 283 for HIV+ subjects [RR of 1.4 (95 % CI 1.3–1.6)]
						Nadir CD4: associated with MIs (RR per 100 cells = 0.88; 95 % CI 0.81–0.96)
						Recent CD4, HIV-RNA, prior ART use, duration of PI and NNRTI: not associated with MIs
Esser et al. [98]	HIV HEART (HIVH) study	Prospective observational	1481 HIV+	7,5 years	To assess the frequency and clinical course of CVE in HIV+ patents by standardized non-invasive CV screening	Advanced clinical and immunological stages:
						Significantly (p < 0.001) associated with higher incidences of CVE (A 17.7 %; B 33.1 %; C 49.2 % and I 3.1 %; II 32.3 %; III 64.6 %)
						No associated with the duration of HIV-infection (per year: HR: 0.91 [0.88–0.94]) and ART (per year: HR: 0.81 [0.79–0.84])
Petoumenos et al. [99]	Data collection on adverse events of anti-HIV Drugs (D:A:D)	Retrospective	24,323 HIV+ men	N/A	To statistically model the relative increased risk of MI, CAD and CVD per year older	Crude MI, CAD and CVD event rates per 1000 person-years increased from 2.29, 3.11 and 3.65 in those aged 40–45 years to 6.53, 11.91 and 15.89 in those aged 60–65 years, respectively
Carballo et al. [100]	Acute myocardial infarction in Switzerland (AMIS) registry plus Swiss HIV cohort study (SHCS) (aggregated data)	Retrospective	Patients who survived an incident MI occurring on or after 1/1/2005: 133 HIV+, 5328 HIV−	1 year	To determine whether HIV infection is a risk factor for worse outcomes in patients who survived an incident MI:	HIV infection associated with a significantly increased risk of all-cause mortality 1 year after incident MI
						No significant differences in recurrent MI (4 [3.0 %] HIV+ and 146 [3.0 %] HIV− individuals, or 1.16, 95 % CI 0.41–3.27)
Klein et al. [10]	Kaiser Permanente California	Retrospective	24,768 HIV+ 257,600 HIV−	15 years	To evaluate changes of MI risk from 1996 to 2011 by HIV status	The adjusted MI RR for HIV status declined from 1.8 in 1996–1999 to 1.0 in 2010–2011

*CAD* coronary artery disease, *CI* confidence interval, *CV* cardiovascular, *CVD* cardiovascular diseases, *CVE* cardiovascular event, *xd* per decade, *HR* Hazard ratio, *MI* myocardial infarction, *N/A* not applicable, *NNRTI* non-nucleoside reverse transcriptase inhibitors, *OR* odds ratio, *PAD* peripheral arterial diseases, *PI* protease inhibitors, *RR* rate ratio, *SCD* sudden cardiac death

### The premature aging of cardiovascular system driven by chronic inflammation and immune activation

Many factors may contribute to the increase of incidence of CVD in HIV infected patients: the traditional determinants of disease (cigarette smoking, diabetes mellitus, dyslipidemia, obesity, systemic hypertension, sedentary lifestyle, stress and family history of coronary artery or vascular disease) appear to be more common than in HIV negative population [11–13]. Moreover alterations of regulation of glucose homeostasis and lipid metabolism and metabolic syndrome are frequently related with cART and are higher in patients previous treated with older antiretroviral drugs [14].

Anyway the excess risk of CVD observed in course of HIV infection cannot be explained only by the factors mentioned above. Several studies have also observed that co-morbidities, like CVD, which are normally witnessed later on in life as a result of natural aging, were increasingly prominent among the HIV-infected population [15, 16]. These observations led to the hypothesis that the cART treated HIV-infected patients experienced a premature and accelerated aging probably driven by side effects of antiretroviral drugs, chronic inflammation and persistent immune activation (Fig. 1).

Undoubtedly a pivotal role in the development of accelerated aging of cardiovascular system and in the pathogenesis of CVD is attributed to the status of immune activation. In fact in HIV patients the levels of proinflammatory cytokines and biomarkers associated to endothelial dysfunction are always higher and they can accelerated the atherosclerosis process. For example monocyte activation caused by microbial translocation drives the releases of soluble CD14 and CD163: the first is associated to increased death and the second with the risk of coronary artery progression and atherosclerosis [17–20]. High levels of these inflammatory markers and of factors of hypercoagulation (i.e. D-dimers, fibrinogen) are linked to systemic clotting and chronic inflammatory damage of vascular endothelium [21]. Also HIV itself is considered responsible of persistent immune activation and endothelial dysfunction in fact it can penetrate into endothelial cells by receptor of CD4-T cells or chemokine receptors pathway. Moreover some HIV viral proteins, such as Gp120 and tumor necrosis-alpha (TNF-α), induce important consequences on the vascular tone, on the adhesion and aggregation of the platelet by the decrease of nitric oxide (NO) levels in the endothelial cell [22–24]. The residual viremia may be the source of harm linked directly to the presence of the virus in course of effective cART, but may also be linked to the maintenance of the process of chronic inflammation and persistent immune activation.

Also Cytomegalovirus (CMV) infection appears to have a role in the premature aging of CV system: in a large Italian cohort CMV/HIV co-infection was associated with the risk of non-AIDS events including CV events independently of other prognostic factors. The potential role of CMV infection in CV disorders could depend by the complex interplay between viral and immunological activation, culminating in cyclical growth, damage and repair of endothelial cells. In fact from a pathogenic point of view, CMV can promote abnormal growth of endothelial cells and pathogenesis of atherosclerosis enhanced by proangiogenic factors including IL-6 and granulocyte macrophage colony stimulating factor [25, 26].

For this reason, the research’s interest is to understand the hidden inflammatory causes of CVD in HIV patients and the first objective is to control the levels of persistent immune activation. In fact, to date, immunological therapeutic strategies and virological interventions are investigated with the aim of controlling the levels of immune activation and then reduce the impact of CVD promoted by chronic inflammation. Table 2 shows the studies, registered on https://www.clinicaltrials.gov and classified as “open”, and provides highlights for the current and future research topics in this area.

**Table 2:** Clinical studies registered on https://www.clinicaltrials.govclassified as “open”, and matched search queries with the following keywords: “HIV”, “cardiovascular”, “inflammation” and/or “immune-activation” (last accession date 19 Dec 2015)

Official title of the study ClinicalTrials.gov Id. Study type and design sponsor	Purpose and description	Primary outcome measures	Secondary outcome measures
A comparison of endothelial function between HIV-infected subjects not receiving anti-retroviral therapy and matched hiv-uninfected con-trol subjects NCT00919724 Observational, case control, prospective Indiana University	The purpose of this study is to determine whether people infected with HIV have worse blood vessel function than people without HIV infection. Specifically, inflammation, immune activation, endothelial activation, and metabolic measures will be compared This study will involve two groups of participants. The first group will consist of people with HIV who are enrolling in two other separate HIV studies (NCT00864916 and NCT00796822), one lasting 8 weeks and the other lasting 48 weeks. The second group will consist of people without HIV who are similar to the first group in terms of age, sex, smoking status, and height	Brachial artery reactivity: the maximum change in brachial artery diameter after induction of reactive hyperemia post-release of vascular occlusion	Inflammatory/endothelial activation markers: (MCP-1, sVCAM-1, IL-6, TNF-a, IP-10, MMP-9, TIMP-1, PAI-1 active, hsCRP) Peripheral blood immune activation: (percentage of CD8 +/CD38 +/HLA-DR+ T cells) Metabolic parameters: (fasting lipoprotein fractions/triglycerides, HOMA-IR)
Biomarkers of inflammation, coagulation, and endothelial function in HIV-infected adults NCT00776412 Observational, prospective NIAID	This study will collect information about markers of inflammation, blood clotting and blood vessel function in HIV-infected adults and healthy volunteers Initially, the study will recruit HIV-infected adults with HIV viremia who are not taking ART and compare their clinical histories and biomarker findings with those from a group of HIV-infected adults with controlled HIV viremia who are receiving ART, and with those from a control group of HIV-negative healthy subjects. Additionally a cohort of HIV-infected adults with poor CD4 + cell recovery despite effective ART, will be enrolled (immunologic non-responder cohort) and for comparison, a control group with similar nadir CD4 counts but with good CD4+ cell recovery on ART	Not provided	Not provided
Open-label, randomized, 24-week pilot study of metformin vs observation for persistent immune activation in chronic HIV infection NCT02383563 Interventional, randomized University of Hawaii	This proposal seeks to assess the impact of 24 weeks of metformin on non-calcified plaques and calcified plaques assessed by coronary CT angiography, and on whether these changes can be explained by metformin-induced phenotypic and secretory changes of monocytes	Coronary plaques by CT angiography change in total numbers of atherosclerotic plaques detected in the coronary arteries	Change in numbers of each monocyte subset Change in monocyte secretory function Change in sub-types of coronary plaques by CT angiography (N° of calcified, non-calcified, mixed atherosclerotic coronary plaques)
Imaging companion study To ACTG A5314: effect of reducing inflammation with low dose methotrexate on inflammatory markers and endothelial function in treated and suppressed hiv infection NCT02312219 Interventional, randomized Massachusetts general hosp	The investigators propose to conduct a time sensitive ancillary imaging study whose overall goal is to determine if treating virologically suppressed, HIV-infected individuals with low-dose methotrexate will reduce inflammation within the arterial wall. arterial FDG uptake provides a measure of inflammation in the artery wall: in fact atherosclerotic inflammation can be non-invasively and reproducibly measured with fluorodeoxyglucose (FDG)-PET/CT imaging, a well-validated quantitative technique that can sensitively detect changes in atherosclerotic inflammation and which has been employed in several multi-center trials to measure changes in arterial inflammation in response to anti-inflammatory treatments	Change in arterial FDG uptake	Change in splenic FDG uptake
Immunologic and inflam-matory factors and cardio-vascular risk in patients with HIV infection or autoimmune diseases NCT01519141 Observational, prospective University of California, San Francisco	The investigators plan to obtain measurement of carotid artery intima media thickness (IMT) using high resolution ultrasound as a noninvasive means for tracking atherosclerotic progression. The investigators will also measure lipid and lipoprotein levels, inflammatory markers, markers of cytomegalovirus (CMV) infection, thrombotic markers, atherogenic lipoproteins, and markers of immune function. immunophenotyping will be performed on freshly collected blood and analyzed by flow cytometry to identify activated T-cells, T-cell turnover, proportions of T-cells, and CMV function. HIV-infected patients will have CD4 count and HIV viral load measured in addition. Patients will also go assessment of endothelial function, endothelial progenitor cells, arterial stiffness as measured using pulse wave tonometry	Increased carotid intima-media thickness (mm) Decreased brachial artery flow-mediated dilatation (%) Increased D-dimer levels (mcg/mL)	Not provided
Effect of reducing inflam-mation with low dose metho-trexate on inflammatory mar-kers and endothelial function in treated and suppressed HIV infection NCT01949116 Interventional, Randomized NIAID and NHLBI	People with HIV infection who are taking antiretroviral therapy may be at risk for cardiovascular disease, which can be caused by inflammation. methotrexate is a medication used to treat inflammation in people with rheumatoid arthritis. This study will evaluate the safety and effectiveness of low-dose methotrexate (LDMTX) at reducing inflammation in HIV-infected adults	Change from baseline to week 24 in brachial artery flow-mediated vasodilation (FMD) (%) (Defined as the maximum FMD (%) calculated from reactive hyperemia (RH) 60 and RH 90 relative to resting artery diameter at baseline) (Other immune-virological and safety endpoints)	Change from baseline to week 12 in brachial artery FMD and brachial artery diameter Change from baseline to week 24 in brachial artery diameter, brachial artery hyperemic flow velocity, levels of high-sensitivity C-reactive protein, IL-6, sCD163, D-dimer, monocyte levels, adhesion and activation indices, and CX3CR1 expression
Effect of IL–1β inhibition on inflammation and cardio-vascular risk NCT02272946 Interventional, randomized University of California, San Francisco	The purpose of this study is to evaluate the effects of IL-1β inhibition on safety, measures of systemic and vascular inflammation and endothelial function (all indicators of cardiovascular risk) in treated and suppressed HIV infected individuals This study will assess the safety and effects of canakinumab on endothelial function (assessed by flow-mediated vasodilation [FMD] of the brachial artery), vascular inflammation (assessed by FDG-PET/CT scanning), key inflammatory markers of cardiovascular disease (CVD) risk (high-sensitivity C-reactive protein [hsCRP]), interleukin-6 (IL-6), soluble CD163 (sCD163), D-dimer, T-cell and monocyte activation in the blood, and size of the HIV reservoir. 20 individuals will receive a single dose of 150 mg canakinumab with follow-up for 18 weeks	Number of adverse events at week 1, 2, 4, 8, 12, 18 as a measure of safety	Change in brachial artery FMD: brachial artery FMD is calculated as the percentage increase in brachial artery diameter with hyperemia induced relative to the resting brachial artery diameter). Change from baseline in FDG uptake assessed by FDG-PET/CT as a measure of vascular inflammation, assessed by FDG-PET/CT scanning Rate of change in inflammatory markers of CVD risk: hsCRP, IL-6, sCD163, D-dimer, T-cell and monocyte activation in the blood, and size of the HIV reservoir
Does rosuvastatin delay progression of atherosclerosis in people with hiv infection at moderate cardiovascular risk? a multicentre rando-mized, double blind placebo-controlled Trial NCT01813357 Interventional, randomized Bayside health	This study is a randomised double blind placebo controlled trial comparing Rosuvastatin with placebo in HIV positive people who are at intermediate cardiovascular risk It is possible that HIV positive people will receive a greater benefit from statins because of their higher baseline levels of inflammation and this study aims to determine what benefit HIV infected people will receive from starting statin therapy earlier then currently recommended Participants will undergo blood tests and ultrasounds of the arteries of the neck (carotid intima media thickness) prior to starting Rosuvastatin and then after 1 and 2 years on the drug to determine what effect it has on markers of inflammation, cholesterol levels and thickness of blood vessels	Progression of carotid intima media thickness. (carotid intima media thickness will be measured by ultrasonography and the change from baseline at 1 and 2 years calculated)	Rates of adverse events (Number of participants with adverse events in total and also the number of participants with adverse events thought secondary to the study medication)
The effects of statin therapy on coronary flow reserve and inflammatory markers in hiv-positive patients NCT02234492 Interventional, efficacy study Ottawa Heart Institute Research Corporation	The purpose of this study is to determine whether the use of rosuvastatin in human immunodeficiency virus (HIV) infected individuals lowers inflammation in blood vessels and improves blood circulation in the small arteries that provide nutrients to the heart muscle	Correlation between coro-nary flow reserve (CFR) and maximum target to background ratio (TBR max). At baseline, corre-lation between CFR by MCE and vascular inflame-mation (TBR max) by FDG-PET/CT will be as-sessed	Changes in CFR as measured by MCE will be evaluated over 6 months Changes in vascular inflammation (TBRmax) as measured by FDG-PET/CT
Myocardial adipose inflam-mation and pericardial adipose volume as markers for coro-nary artery disease in HIV positive patients NCT02399384 Observational, case control University of Cincinnati	The investigators propose to correlate 1) cardiac MRI pericardial adipose volume, 2) the presence of pericardial monocytes and 3) circulating immune biomarkers in persons with and without CHD and HIV infection compared to seronegative controls with known CHD. The investigators aim to test the hypothesis that higher amounts of pericardial fat deposition and increased presence of monocytes within this adipose tissue are associated with underlying coronary artery disease in persons with HIV infection as measured by cardiac MRI	Pericardial adipose tissue volume	Adipose spin spin relaxivity as measured by T2 star time
Evaluating the use of pitavastatin to reduce the risk of cardiovascular disease in HIV-infected adults (REPRIEVE) NCT02344290 Interventional National Institute of Allergy and Infectious Diseases (NIAID)	This study will evaluate the use of pitavastatin to reduce the risk of CVD in adults infected with HIV who are on antiretroviral therapy (ART). This study will enroll adults infected with HIV who are on any ART regimen (ART is not provided by the study) for at least 6 months before study entry considered low-to-moderate risk using the 2013 American College of Cardiology (ACC)/American Heart Association (AHA) guideline thresholds for recommended statin initiation. Total study duration will be approximately 72 months from the time the first participant is enrolled Participants will be randomly assigned to receive 4 mg of pitavastatin or placebo once a day for the entire time they are enrolled in the study Some participants will have the option of enrolling in a substudy (effects of pitavastatin on coronary artery disease and inflammatory biomarkers: mechanistic substudy of REPRIEVE [A5333 s]). The substudy will evaluate the effect of pitavastatin on the progression of non-calcified coronary atherosclerotic plaque (NCP) and inflammatory biomarkers in adults infected with HIV. Participants in the substudy will attend study visits at study entry and months 4 and 24. The visits will include questionnaires and assessments, a blood collection, and a coronary computed tomography angiography (CCTA)	Time to the first event of a composite of major cardiovascular events (Includes atherosclerotic or other CVD death, nonfatal myocardial infarction, unstable angina hospital-lization, coronary or peripheral arterial revascu-larization, nonfatal stroke or transient ischemic attack (TIA), urgent peripheral arterial disease (PAD) ischemic event)	Time to the first of each individual component of the primary endpoint Time to death (all-cause mortality) Time to death (all-cause mortality) and/or major adverse cardiovascular events (MACE) Time to any (composite) or each (individual) of the following clinical diagnoses (Non AIDS-defining cancers; AIDS-defining events; initiation of dialysis or renal transplantation; cirrhosis, or hepatic decompensation requiring hospitalization) Calculated fasting LDL and HDL cholesterol level Time to any of the following adverse events (Serious adverse event, incident diabetes mellitus (DM), grade 3 or 4 ALT, or grade 3 or 4 myopathy)

*ART* antiretroviral therapy, *NIAID* National Institute of Allergy and Infectious Diseases, *NHLBI* National Heart, Lung, and Blood Institute

### Current trends and challenges in evaluation of CV risk in HIV positive patients

As previous described, an increase of morbidity and mortality related to clinical and sub-clinical CVD in HIV patients are reported. An important paradigm in the comprehension of this increased incidence is to understand the role of traditional risk factors. Evaluating the HIV cohorts, numerous data suggest that the prevalence of “traditional” risk factors is higher in HIV subjects if compared with general population. Triant et al. [27] observed greater incidence of hypertension (HIV: 21.2 vs. non-HIV: 15.9 %; p < 0.001), diabetes (HIV: 11.5 vs. non-HIV: 6.6 %; p < 0.0001), and dyslipidemia (HIV: 23.3 vs. non-HIV: 17.6 %; p < 0.0001) in HIV positive patients than in the controls. In particular, the serum triglyceride levels are higher in HIV population respect to controls [28]. Moreover, triglycerides work in a synergic way with both infection and cART: in fact, triglycerides levels increase when signs and symptoms of AIDS appear but also their serum levels are raised by protease inhibitors therapy [29].

However the high prevalence of “traditional” risk factors do not seem to be sufficient to explain the increased cardiovascular risk observed in the HIV positive population and the cardiovascular risk assessment based on conventional risk prediction models (i.e. Framingham system, PROCAM, SCORE) does not offer a good predictive value for the HIV-positive population [30]. In fact cardiovascular risk scores were built for general population, and were not validated in the HIV positive population. Probably the factors that most affect the failure in estimating the cardiovascular risk in HIV-positive patients are HIV infection itself and the premature aging of CV system driven by chronic inflammation and immune activation: in this sense the virus may be considered “the missing factor” essential for a correct evaluation of the overall risk [31].

Given the inadequacy of the risk estimation algorithms available, recently new models of risk prediction, specific for HIV-positive patients, were designed: for example Friis-Moller et al. [31] proposed the Data Collection on Adverse Effects of Anti-HIV Drugs (D:A:D) study equation in which age, sex, systolic blood pressure, serum cholesterol total and HDL-cholesterol level, diabetes, smoking status, family history of CVD, current use of abacavir, indinavir, or lopinavir; and the number of years on indinavir or lopinavir were the variables included. This cardiac risk model was created based upon data from the large cohort of HIV-infected patients who were followed longitudinally for cardiac events and performed better than the Framingham risk score among patients in that cohort. Moreover increased C-reactive protein (CRP) levels, uncontrolled HIV viral load at time of CV event and slower immunologic response were found to be associated with increased CVD risk in D:A:D: cohort [32]. Anyway a recent study of Thompson-Paul et al. [33] evaluating the D:A:D score and comparing with traditional algorithm for CV risk evaluation in HIV negative patients, highlighted that also the use of this algorithm was associated with the underestimation of CV risk. The authors concluded underlying that to better estimate CV risk in HIV-infected persons, additional risk factors, such as immunologic or virologic status may need to be considered.

The failure of traditional methods of CV risk assessment has made it necessary to evaluate new methods to better understand the risk of CVD in HIV population.

Among the inflammatory biomarkers of CV risk, several have been proposed for diagnostic use, including IL-6, D-dimer, MMP-9, and high-sensitivity CRP (hsCRP). In HIV seronegative patients, hsCRP has emerged as the most useful because it has been proven to add risk to the other factors described within the Framingham cohort. Anyway for the time being, the meaning of hsCRP cannot be relied upon in the same manner in patients with HIV infection, in fact the results of studies in this setting are conflicting [34, 35].

In the field of diagnostic imaging, carotid intima-media thickness (cIMT), brachial ankle pulse wave velocity and coronary artery calcium (CAC) score are innovative indicators for assessment of subclinical atherosclerosis and could give information about the development of CVD in patient without history of atherosclerotic diseases. These cardiovascular disease risk surrogate markers, are currently relegated to a secondary role, anyway their results should be considered complementary, and not alternative, to the information provided by CVD risk scores. In fact CVD risk assessment algorithms were not developed to predict coronary or carotid atherosclerosis, but rather cardiovascular events depending on the algorithm. For example baseline levels and change in levels of subclinical atherosclerosis, assessed by cIMT, could provide additional informations to determine how intensively to intervene on lifestyle and whether medication in correspondence with individual’s cardiovascular risk factors is indicated [36–40].

Therefore in the last years the search for an optimal algorithm for estimating cardiovascular risk has been partly replaced by the search for exams increasingly able to estimate directly the presence of subclinical atherosclerotic lesions. The prevention is therefore increasingly moving towards the ability to diagnose the presence of silent atherosclerotic lesions able to progress and to determine a significant symptoms in asymptomatic subjects. On the basis of the above considerations the diagnostic applications of molecular imaging techniques becomes always more important in the detection of atherosclerotic plaques. The imaging by 18-fluoredeoxyglucose positron emission tomography (PET) and single photon emission computed tomography (SPECT) are considered important to identify activated macrophages infiltrating the arterial wall. Moreover both PET and SPECT provide information about the risk of plaque rupture and furthermore evaluate the vulnerability of itself. Recently Knudsen et al. conducted a prospective cross sectional study in which 56 HIV positive patients and 25 controls were scanned using 82Rb PET to obtain the stress flow (MFR). The results obtained were stratified in: low <1.5, borderline >1.5–2 or normal >2 and the Author did not observe difference between HIV positive and negative controls [41]. The data about the use of PET and SPECT in HIV population are not enough to conclude the useful of that in the real life. On the other hand the utility of imaging techniques in the detection of subclinical atherosclerotic plaques was reported in a cross-sectional study using dual-source CT (MDCT) coronary angiography conducted in HIV-infected subjects with low CV risk: MDCT showed an unexpected, age-associated high rate of significant coronary stenosis requiring coronary angiography in 29.1 % of asymptomatic HIV positive subjects enrolled. On the basis of these findings authors suggested that MDCT may be appropriate for CVD screening programs in HIV positive population [42].

Detection of subclinical atherosclerosis by noninvasive tests such as CAC score, cIMT, MDCT, and PET may improve risk prediction above that of established risk scoring models. Moreover interesting results are expected by promising innovative techniques, such as 3D quantification of carotid plaque by ultrasound (as in the Bioimage study), which are still waiting to be properly tested in HIV-positive population.

However, despite the interesting results obtained, more studies are need to investigate if diagnostic imaging exams can be used to guide patient screening, management and therapy in HIV setting.

### Clinical impact of inflammation on cardiovascular system: from subclinical atherosclerosis to complications of coronary revascularization

As previously reported, HIV-infected patients receiving combination antiretroviral therapy may experience metabolic complications (i.e. dyslipidemia, impaired glucose metabolism and abnormal body fat distribution), potentially increasing their risk of cardiovascular diseases. In fact HIV infection is associated with a marked rise in the frequency of coronary heart disease and with accelerated coronary atherosclerosis and vasculopathy.

The progression from subclinical atherosclerosis to symptomatic disease is estimated to be higher and earlier in HIV patients than in general population. In fact HIV patients have a high burden of subclinical atherosclerosis including an increased amount of non-calcified coronary plaque compared with HIV-uninfected individuals independently by cardiovascular risk.

On these basis, recently, an increasing number of HIV patients undergo invasive cardiovascular procedures as a result of acute cardiovascular events or severe atherosclerotic disease. Although percutaneous coronary intervention (PCI) frequently used to treat coronary artery disease in HIV, little is known regarding the outcomes and the effects of immune activation on the this procedure.

In literature an observational study on 50 HIV infected patients undergoing PCI found that this procedure is a safe treatment strategy of coronary revascularization in HIV positive patients without significant differences in terms of clinical restenosis from the control population [43].

On the other hand Martín-Reyes et al. [44] reported that in-hospital prognosis in patients with HIV, undergoing PCI, was worse than in control subjects: in fact HIV-positive patients presented a lower rate of PCI success (75 vs. 85 %) respect HIV-negative patients. Moreover Segev et al. [45] analyzing the long-term outcome of PCI in 12 HIV-infected patients, observed that 58.3 % of subjects suffered from severe clinical and/or angiographic restenosis requiring additional interventions or causing severe angina pectoris. They concluded that HIV-infected patients should be considered as high risk group and treated routinely with drug-eluting stents but they did not given any pathogenic interpretation of their observations. Although HIV-infected patients have a higher incidence of post-PCI ischemic events, restenosis, and stent thrombosis, Matetzky underlined that the intermediate-term mortality is low [46].

At this moment few data are available regarding the pathogenesis of restenosis after coronary revascularization in HIV positive patients. It is possible that the concomitant presence of traditional risk factors, assumption of antiretroviral therapy and his metabolic complications contribute to the restenosis events after PCI in HIV-positive population. However, although the presence of these well-known reasons of atherogenesis, the rapidity of progression of atherosclerosis and restenosis process seems linked principally to chronic inflammation and excess immune activation in HIV-infected patients [47–49]. In fact chronic HIV infection is characterized by multifaceted systemic immune activation, including increased frequencies of activated T-cells and increased turnover of T-cells that correlate directly with disease progression [50]. Moreover a previous study have described other conditions (coronary allograft vasculopathy, native atherosclerosis) associated with an inappropriate inflammatory reactivity that may predispose to re-stenosis after PCI [51]. For these reasons therapies that reduce immune activation may be of benefit, particularly for such individuals [50, 52]; moreover a strictly monitoring of coronary atherosclerosis is required in management of HIV-infected patients with concomitant non-traditional risk factors for intense immune-activation.

In summary contrasting results are reported in the literature on the outcome of coronary revascularization; moreover the coronary revascularization with PCI and stents seems to be influenced by the excess of HIV related inflammation and immune activation [53]. Large clinical studies are needed to clarify aspects of the matter is not yet resolved.

### New therapeutic insights in minimizing the risk of cardiovascular disease

As previously highlighted, HIV-infected patients experienced a premature and accelerated aging probably driven by chronic inflammation and persistent immune activation. For this reason currently immunological therapeutic strategies and virological interventions are investigated with the aim of controlling the levels of immune activation and then reducing the impact of CVD promoted by chronic inflammation.

An innovative immunological/pharmacological intervention, granted by experience in HIV negative populations, is based on use of statins. These class of drugs, widely used for primary prevention of atherosclerotic CVD, are known to decrease cholesterol level as well as inflammation in HIV seronegative patients. For example in the JUPITER trial rosuvastatin significantly reduced all-cause mortality and in particular the incidence of major cardiovascular events in healthy persons without hyperlipidemia but with elevated hsCRP levels, a well-known marker of inflammation [54]. Statins will emerge also as potential components of the therapeutic armamentarium for chronic inflammatory and autoimmune pathologies which share some pathogenetic aspects concerning chronic inflammation with HIV infection [55]. In fact statin therapy has been shown to reduce disease severity in patients with rheumatoid arthritis (RA) and to improve endothelium-dependent vasodilation in patients with RA and systemic lupus erythematosus (SLE) [56–60]. In systemic inflammatory diseases (such as RA, SLE, inflammatory bowel diseases, psoriasis, spondyloarthritis and others) multiple factors, including tumor necrosis factor-α (TNF-α), circulating inflammatory cytokines, reactive oxygen species, autoantibodies, and oxidized low density lipoprotein (LDL) directly and indirectly activate endothelial cells, increased endothelial permeability, increased leukocyte adhesion and generation of a pro-thrombotic state. In this case statins could have a potential role in modifying initiation and amplification of immune inflammatory responses and their anti-inflammatory activity is linked to different mechanisms including (but not limited) the decrease in chemotaxis of monocytes and macrophages, the lipopolysaccharide (LPS) mediated release of TNF-α, and the activation of NO synthase [61–64].

The efficacy and safety of different statins for HIV-infected individuals in the primary prevention setting remain to be established. Also the effect of statins use on overall mortality in HIVpositive patients remains controversial [65, 66].

A recent meta-analysis, including 18 clinical studies with 736 HIV-positive patients under cART treated with statins in the primary prevention, showed that this therapy significantly lowers plasma total cholesterol (TC) and low density lipoprotein (LDL) levels. In particular rosuvastatin 10 mg and atorvastatin 10 mg provided the largest reduction in TC levels while atorvastatin 80 mg and simvastatin 20 mg had the largest reduction in LDL [67].

Moreover atorvastatin and rosuvastatin appear to reduce non-calcified coronary plaque volume and slow progression of carotid intima-media thickness in HIV positive patients under cART [68].

Conversely the effects of statins on inflammation and are less conclusive at the moment: a recent study, evaluating the effect of rosuvastatin on markers of immune activation in treatment-naive HIV-patients, showed that this molecule had a small but significant positive effect on CD4/CD8 T cell ratio, but not influenced other activation markers (neopterin, soluble Toll-like receptor (TLR) 2, sTLR4, interleukin (IL)-6, IL-1Ra, IL-18, D-dimer, hsCRP) [69]. Interesting the protective effect of statins on CV system is related not only to their anti-inflammatory activity but also to a control of oxidative stress: in fact oxidative stress plays a significant role in atherosclerosis development and is also elevated in HIV+ patients due to excessive production of free radicals that play a major role in oxidative modification in LDL particle. Serum oxidized LDL levels may contribute to monocyte activation, are associated with clinical manifestations of atherosclerosis, decreases in response to statin therapy and relates independently to reductions in coronary plaque in patients with HIV. Statins may reduce oxidative stress and subsequently subclinical vascular disease in HIV but further study in the context of HIV disease is warranted [70–72].

Considered the importance of the topic and the still existing doubts, the US National Institutes of Health (NIH) recently launched the REPRIEVE trial to definitively assess the efficacy of statins as a primary prevention strategy for CVD in this at-risk population. This clinical study launched in 2015 is the largest ongoing trial of HIV-related CVD to date (see Table 2) [73].

Some concerns are the potential side effects described for these class of drugs. i.e. statin use was found associated with a modestly increased risk of incident diabetes mellitus in HIV Outpatient Study (HOPS) participants [74]. Also severe rhabdomyolysis, hepatotoxicity and acute renal failure were described in HIV positive population [75–77]. Concerns are also the potential numerous drug–drug interactions with cART dependent on statins’ pharmacokinetic profile: i.e. simvastatin and lovastatin, metabolized through cytochrome P450 (CYP) 3A, have the significant potency for drug–drug interaction with potent CYP3A inhibitors such as ritonavir- or cobicistat-boosted HIV-protease inhibitors (PIs). Less potent drug–drug interactions have been reported for atorvastatin although it is also a CYP3A substrate. Non-CYP3A-dependent statin concentrations are also affected when co-administered with PIs, mainly through interaction with OATP1B1 [78, 79].

Interesting pitavastatin, a newer statin that does not have substantial interactions with antiretroviral drugs, will be tested in the REPRIEVE trial. At the present time, however, many aspects of potential drug interactions remain to be clarified. Despite all the warnings described, the overall use of statins in HIV population is associated with low rates of adverse events and the mean discontinuation rate reported in meta-analysis and attributable to these problems was 0.12 per 100 person-years [95 % CI (0.05, 0.20)] [67].

The possibility that residual immune activation (and the consequent inflammation) is related to persistent virus replication has suggested that more potent anti-retroviral regimen (i.e., ART “intensification”) may be more effective in reducing the HIV-associated immune activation. In one study intensification was carried out for 10 weeks with cART regimens (atazanavir/ritonavir, lopinavir/ritonavir, efavirenz) consisting of drugs that were not used previously in the enrolled patients. Since the median levels of viremia were not significantly different between pre-intensification and post-intensification period, the authors concluded that the levels of residual virus replication, and thus immune activation, may depend on the size of stable reservoir compartments that are established prior to the initiation of cART [80]. Another study of Llibreet et al. [81] assigned randomly 69 patients with undetectable levels of viraemia for more than 1 year to treatment groups in which 45 intensify therapy with raltegravir and 24 continue the same therapy for 48 weeks. The author observed after 24 weeks in the group of intensification therapy a reduction of the size of the reservoirs and of the levels of immune activation (measured as fraction of CD8+ T-cells expressing CD38+ and/or HLA-DR). Further studies on the effects of cART intensification on the residual immune activation will be needed to ascertain whether a complete recovery of the “ideal” pre-HIV infection immune system function can be achieved through virological interventions alone. In absence of conclusive results in this research field, the alternative possibilities, i.e. specific immune-based interventions, will be necessary to try to protect HIV infected individuals by damages of immune activation. In this sense we know that the most important source of the status of generalized inflammation is attributed to the breakdown in the integrity of the gut mucosa with the increase of microbial translocation and the transition of the microbial products in the systemic circulation. For this reason different trials were conducted to explore the possibility of intervention on this source of chronic inflammation. In a recent study in order to enhance GI tract immunity, we recruited and treated 20 HIV-infected humans with cART supplemented with probiotics and followed inflammation and immunological parameters. We observe that cART did not normalize the levels of immune activation in HIV positive patients, anyway inflammation and markers of microbial translocation were significantly reduced with probiotic supplementation. Patients enrolled showed a clear and statistically significant reduction in the levels of immune activation on CD4 T-lymphocytes, for both markers CD38 and HLA-DR and their simultaneous expression, LBP and hsCRP plasma levels after probiotic diet supplementation. In conclusion this study, like other recent reports [82–85], evidenced that supplementing cART with probiotics in HIV-infected individuals may improve GI tract immunity and thereby mitigate inflammatory sequelae, ultimately improving prognosis [86].

Another potentially interesting therapeutic concept for the reduction of high levels of inflammation and to control CVD is to use non pharmacological tools of intervention such as physical activity. Several published studies indicate that physical activity could represent a beneficial non-pharmacological intervention to reduce chronic inflammation. Currently available data are limited, nonetheless increasing evidence suggests that the introduction of regular physical exercise in the clinical management of HIV-infected individuals may have a significant therapeutic impact in reducing inflammation and CVD. For example Longo et al. [87] conducted a longitudinal study on 50 ART-treated HIV-infected patients with sedentary life style to evaluate the effect of 12 weeks of moderate intensity exercise on parameters of immune activation and metabolic profile. At the enrollment and at 12 weeks all participants were assessed by a 6-min walking test and 1-RM, DEXA, metabolic and immune-virologic parameters, and markers of immune activation (IL-6, d-Dimer, sCD14 and IL-18). The authors found a significant improvement of both fitness and immune activation at the week-12 time point.

On the basis of these evidences, several authors proposed that regular physical exercise should be further studied as a potential anti-inflammatory, non-pharmacological approach to be used to treat HIV residual disease and non-AIDS-defining illnesses in cART-treated HIV-infected individuals [88–94].

Likewise, lifestyle interventions are another non pharmacological tool reputed able to reduce cardiovascular risk in general population: nevertheless a recent randomized pilot study with a follow-up 36 months evidenced that a multidisciplinary lifestyle intervention resulted in a slight improvement in some cardiovascular risk factors and did not prevent cIMT progression in HIV-infected patients with Framingham scores >10 % [95]. Further studies are required to confirm the importance of all these data.

## Conclusions

In cART treated HIV-infected individuals with undetectable viremia the level of immune activation is dramatically reduced compared to baseline (i.e., pre-treatment) but rarely goes back to normal levels. This immune activation is associated with long-term sequelae of HIV infection such as accelerated atherosclerosis, neurological disease, and several other conditions that may modify the quality of life of HIV-infected subjects. In addition, the residual viral replication appears to be a uncontrolled determinant of the levels of immune activation in HIV patients treated with cART.

The goal of practitioners is changed in the last years: in fact if initially it was to reach undetectable plasma levels of HIV viremia, now it is to prevent the premature aging and improve the quality of life of all patients. For this reason it becomes necessary to control the hidden effects of HIV, to develop safe antiretroviral regimen and discover new scores to predict the non AIDS related manifestations.

## References

[1] Chastain DB, Henderson H, Stover KR (2015). Epidemiology and management of antiretroviral-associated cardiovascular disease. Open AIDS J..

[2] Favre D, Mold J, Hunt PW, Kanwar B, Loke P, Seu L (2010). Tryptophan catabolism by indoleamine 2,3-dioxygenase 1 alters the balance of TH17 to regulatory T cells in HIV disease. Sci Transl Med.

[3] d’Ettorre G, Douek D, Paiardini M, Ceccarelli G, Vullo V (2012). Microbial translocation and infectious diseases: what is the link?. Int J Microbiol.

[4] Vyboh K, Jenebian MA, Mehraj V, Routy JP (2015). HIV and GUT microbiota, partners in crime: breaking the vicious cycle to unearth new therapeutic targets. J Immunol Res.

[5] Ancuta P, Monteiro P, Sekaly RP (2010). TH17 lineage commitment and HIV-pathogenesis. Curr Opin HIV AIDS.

[6] Shaw JM, Hunt PW, Critchfield JW, McConnell DH, Garcia JC, Pollard RB (2011). Increased frequency of regulatory T cells accompanies increased immune activation in rectal mucose of HIV-positive noncontrollers. J Virol.

[7] d’Ettorre G, Paiardini M, Ceccarelli G, Silvestri G, Vullo V (2011). HIV-associated immune activation: from bench to bedside. AIDS Res Human Retrovir.

[8] Freiberg MS, Chang CC, Kuller LH, Skanderson M, Lowy E, Kraemer KL (2013). HIV infection and the risk of acute myocardial infarction. JAMA Intern Med..

[9] Silverberg MJ, Leyden WA, Xu L, Horberg MA, Chao CR, Towner WJ (2014). Immunodeficiency and risk of myocardial infarction among HIV-positive individuals with access to care. J Acquir Immune Defic Syndr.

[10] Klein DB, Leyden WA, Xu L, Chao CR, Horberg MA, Towner WJ (2015). Declining relative risk for myocardial infarction among HIV-positive compared with HIV-negative individuals with access to care. Clin Infect Dis.

[11] Lifson AR, Nehaus J, Arribas JR, van den Berg-Wolf M, Labriola AM, Read TR (2010). Smoking-related health risks among persons with HIV in strategies for management of antiretroviral therapy clinical trial. Am J Public Health.

[12] Friis-Moller N, Weber R, Reiss P, Thiebaut R, Kirk O, d’Arminio Monforte A (2003). Cardiovascular disease risk factors in HIV patients association with antiretroviral therapy. Results from the DAD study. AIDS.

[13] Brown TT, Cole SR, Li X, Kingsley LA (2005). Antiretroviral therapy and the prevalence and incidence of diabetes mellitus in the multicenter AIDS cohort study. Arch Intern Med.

[14] d’Ettorre G, Ceccarelli G, Zaccarelli M, Ascoli-Bartoli T, Bianchi L, Bellelli V, et al. Impact of switching from lopinavir/ritonavir to boosted and un-boosted atazanavir on glucose metabolism: ATAzanavir & GLUcose metabolism (ATAGLU) study. Int J STD AIDS. 2015. 10.1177/0956462415590724 26068963

[15] Deeks SG, Phillips AN (2009). HIV infection, antiretroviral treatment, ageing, and non-AIDS related morbidity. BMJ.

[16] Guaraldi G, Orlando G, Zona S, Menozzi M, Carli F, Garlassi E (2011). Premature age-related comorbidities among HIV-infected persons compared with the general population. Clin Infect Dis.

[17] Burdo TH, Lo J, Abbara S, Wei J, DeLelys ME, Preffer F (2011). Soluble CD163, a novel marker of activated macrophages, is elevated and associated with noncalcified coronary plaque in HIV-infected patients. J Infect Dis.

[18] Longenecker CT, Funderburg NT, Jiang Y, Debanne S, Storer N, Labbato DE (2013). Markers of inflammation and CD8 T-cell activation, but not monocyte activation, are associated with subclinical carotid artery disease in HIV-infected individuals. HIV Med.

[19] Martin GE, Gouillou M, Hearps AC, Angelovich TA, Cheng AC, Lynch F (2013). Age-associated changes in monocyte and innate immune activation markers occur more rapidly in HIV infected women. PLoS ONE.

[20] Fitch KV, Srinivasa S, Abbara S, Burdo TH, Williams KC, Eneh P (2013). Noncalcified coronary atherosclerotic plaque and immune activation in HIV-infected women. J Infect Dis.

[21] Arildsen H, Sørensen KE, Ingerslev JM, Østergaard LJ, Laursen AL (2013). Endothelial dysfunction, increased inflammation, and activated coagulation in HIV-infected patients improve after initiation of highly active antiretroviral therapy. HIV Med.

[22] Jiang J, Fu W, Wang X, Lin PH, Yao Q, Chen C (2010). HIV gp120 induces endothelial dysfunction in tumour necrosis factor-alpha-activated porcine and human endothelial cells. Cardiovasc Res.

[23] Graham SM, Mwilu R, Liles WC (2013). Clinical utility of biomarkers of endothelial activation and coagulation for prognosis in HIV infection: a systematic review. Virulence.

[24] López M, San Román J, Estrada V, Vispo E, Blanco F, Soriano V (2012). Endothelial dysfunction in HIV infection–the role of circulating endothelial cells, microparticles, endothelial progenitor cells and macrophages. AIDS Rev.

[25] Lichtner M, Cicconi P, Vita S, Cozzi-Lepri A, Galli M, Lo Caputo S, ICONA Foundation Study (2015). Cytomegalovirus coinfection is associated with an increased risk of severe non-AIDS-defining events in a large cohort of HIV-infected patients. J Infect Dis.

[26] Barrett L, Fowke KR, Grant MD (2012). Cytomegalovirus, aging, and HIV: a perfect storm. AIDS Rev.

[27] Triant VA, Lee H, Hadigan C, Grinspoon SK (2007). Increased acute myocardial infarction rates and cardiovascular risk factors among patients with human immunodeficiency virus disease. J Clin Endocrinol Metab.

[28] Olalla J, Crespo E, De la Torre J, Sempere M, Del Arco A, Prada JL (2015). Factors related to NT-proBNP levels in HIV patients aged over 40 years. AIDS Res Ther.

[29] Blázquez D, Ramos-Amador JT, Saínz T, Mellado MJ, García-Ascaso M, De José MI (2015). Lipid and glucose alterations in perinatally-acquired HIV-infected adolescents and young adults. BMC Infect Dis.

[30] Ceccarelli G, d’Ettorre G, Vullo V (2013). The challenge of cardiovascular diseases in HIV-positive patients: it’s time for redrawing the maps of cardiovascular risk?. Int J ClinPract.

[31] Friis-Moller N, Thiebaut R, Reiss P, Weber R, Monforte AD, De Wit S (2010). Predicting the risk of cardiovascular disease in HIV-infected patients: the data collection on adverse effects of anti-HIV drugs study. Eur J Cardiovasc Prev Rehabil.

[32] Markowicz S, Delforge M, Necsoi C, De Wit S (2014). Cardiovascular risk evaluation of HIV-positive patients in a case-control study: comparison of the D:A: D and Framingham equations. J Int AIDS Soc.

[33] ThompsonPaul AM, Lichtenstein KA, Armon C. Cardiovascular disease risk prediction in the HIV Outpatient Study (HOPS). Conference on retroviruses and opportunistic infections. Seattle: Abstract Number: 747; 2015. Available from: http://www.croiconference.org/sessions/cardiovascular-disease-risk-prediction-hiv-outpatient-study-hops

[34] Libby P, Ridker PM, Hansson GK (2009). Leducq transatlantic network on atherothrombosis. Inflammation in atherosclerosis: from pathophysiology to practice. J Am Coll Cardiol.

[35] Fichtenbaum CJ (2011). Inflammatory markers associated with coronary heart disease in persons with HIV infection. Curr Infect Dis Rep.

[36] Volpe GE, Tang AM, Polak JF, Mangili A, Skinner SC, Wanke CA (2013). Progression of carotid intima-media thickness and coronary artery calcium over 6 years in an HIV-infected cohort. J Acquir Immune Defic Syndr.

[37] Mangili A, Gerrior J, Tang AM, O’Leary DH, Polak JK, Schaefer EJ (2006). Risk of cardiovascular disease in a cohort of HIV-infected adults: a study using carotid intima-media thickness and coronary artery calcium score. Clin Infect Dis.

[38] Longenecker CT, Jiang Y, Orringer CE, Gilkeson RC, Debanne S, Funderburg NT (2014). Soluble CD14 is independently associated with coronary calcification and extent of subclinical vascular disease in treated HIV infection. AIDS.

[39] Olalla J, Salas D, De La Torre J, Del Arco A, Prada JL (2011). García Alegría [Ankle-brachial index in the assessment of cardiovascular risk among HIV infected patients. J Rev Med Chil.

[40] Qaqa AY, Debari VA, El-Kersh K, Sison R, Isbitan A, Mohammad N, Slim J, Perez G, Shamoon FE (2012). Epidemiologic aspects of abnormal ankle brachial index in the HIV infected population. Int Angiol.

[41] Knudsen A, Christensen TE, Ghotbi AA, Hasbak P, Lebech AM, Kjær A (2015). Normal myocardial flow reserve in HIV-infected patients on stable antiretroviral therapy: a cross-sectional study using Rubidium-82 PET/CT. Medicine (Baltimore).

[42] d’Ettorre G, Francone M, Mancone M, Ceccarelli G, Ascarelli A, Vullo F (2012). Significant coronary stenosis detected by coronary computed angiography in asymptomatic HIV infected subjects. J Infect.

[43] Boccara F, Teiger E, Cohen A, Ederhy S, Janower S, Odi G, Di Angelantonio E, Barbarini G, Barbaro G (2006). Percutaneous coronary intervention in HIV infected patients: immediate results and long term prognosis. Heart.

[44] Martín-Reyes R, Galeote G, Moreno R, Sánchez-Recalde A, López De Sá E, López-Sendón JL (2010). Percutaneous coronary intervention in patients infected with human immunodeficiency virus admitted with an acute coronary syndrome: Case-control study. Med Clin (Barc).

[45] Segev A, Cantor WJ, Strauss BH (2006). Outcome of percutaneous coronary intervention in HIV-infected patients. Catheter Cardiovasc Interv.

[46] Matetzky S, Domingo M, Kar S, Noc M, Shah PK, Kaul S, Daar E, Cercek B (2003). Acute myocardial infarction in human immunodeficiency virus-infected patients. Arch Intern Med.

[47] De Lorenzo F, Collot-Teixeira S, Boffito M, Feher M, Gazzard B, McGregor JL (2008). Metabolic-inflammatory changes, and accelerated atherosclerosis in HIV patients: rationale for preventative measures. Curr Med Chem.

[48] van Leuven SI, Sankatsing RR, Vermeulen JN, Kastelein JJ, Reiss P, Stroes ES (2007). Atherosclerotic vascular disease in HIV: it is not just antiretroviral therapy that hurts the heart!. Curr Opin HIV AIDS..

[49] Libby P, Okamoto Y, Rocha VZ, Folco E (2010). Inflammation in atherosclerosis: transition from theory to practice. Circ J.

[50] Murray SM, Down CM, Boulware DR, Stauffer WM, Cavert WP, Schacker TW (2010). Reduction of immune activation during chronic HIV infection with chloroquine therapy. J Virol.

[51] Hognestad A, Endresen K, Wergeland R, Mellembakken JR, Mollnes TE, Omland T (2005). Inflammatory response and re-stenosis after percutaneous coronary intervention in heart transplant recipients and patients with native atherosclerosis. J Heart Lung Transplant.

[52] Gonzalez VD, Falconer K, Blom KG, Reichard O, Mørn B, Laursen AL (2009). High levels of chronic immune activation in the T-cell compartments of patients coinfected with hepatitis C virus and human immunodeficiency virus type 1 and on highly active antiretroviral therapy are reverted by alpha interferon and ribavirin treatment. J Virol.

[53] Ceccarelli G, d’Ettorre G, Mancone M, Francone M, Vullo V (2011). Accelerated coronary atherosclerosis after execution of percutaneous coronary intervention in patient with HIV/HCV coinfection: case report and review of the literature. Cardiovasc Revasc Med..

[54] Ridker PM, Danielson E, Fonseca FAH, Genest J, Gotto AM, Kastelein JJ (2008). Rosuvastatin to prevent vascular events in men and women with elevated C-reactive protein. N Engl J Med.

[55] Grover S (2003). Role of statins in autoimmune disease. J Indian Rheumatol Assoc.

[56] Maki-Petaja KM, Booth AD, Hall FC, Wallace SM, Brown J, McEniery CM, Wilkinson IB (2007). Ezetimibe and simvastatin reduce inflammation, disease activity, and aortic stiffness and improve endothelial function in rheumatoid arthritis. J Am Coll Cardiol..

[57] Kanda H, Hamasaki K, Kubo K, Tateishi S, Yonezumi A, Kanda Y, Yamamoto K, Mimura T (2002). Antiinflammatory effect of simvastatin in patients with rheumatoid arthritis. J Rheumatol.

[58] McCarey DW, McInnes IB, Madhok R, Hampson R, Scherbakova O, Ford I, Capell HA, Sattar N (2004). Trial of atorvastatin in rheumatoid arthritis (TARA): double-blind, randomised placebo-controlled trial. Lancet.

[59] Okamoto H, Koizumi K, Kamitsuji S, Inoue E, Hara M, Tomatsu T, Kamatani N, Yamanaka H (2007). Beneficial action of statins in patients with rheumatoid arthritis in a large observational cohort. J Rheumatol.

[60] Sheng X, Murphy MJ, Macdonald TM, Wei L (2012). Effectiveness of statins on total cholesterol and cardiovascular disease and all-cause mortality in osteoarthritis and rheumatoid arthritis. J Rheumatol.

[61] Palinsk W (2001). New evidence for beneficial effects of statins unrelated to lipid lowering. Arterioscler Thromb Vasc Biol.

[62] Weitz-Schimdt G, Welzenbach K, Brinkmann V, Kamata T, Kallen J, Bruns C (2001). Statins selectively inhibit leucocyte function antigen-1 by binding to a novel regulatory integrin site. Nat Med.

[63] Wong B, Lumma WC, Smith AM, Sisko JT, Wright SD, Cai TQ (2001). Statins suppress THP-1 cell migration and secrection of matrix metalloproteinase 9 by inhibiting geranylgeranylation. J LeukocBiol.

[64] Steyers CM, Miller FJ (2014). Endothelial dysfunction in chronic inflammatory diseases. Int J Mol Sci.

[65] Drechsler H, Zhang S, Maalouf N, Cutrell J, Tebas P, Bedimo R. (2013) Impact of statin exposure on mortality and non-AIDS complications in HIV patients on HAART. 20th Conference on retroviruse and opportunistic infections (CROI). Atlanta; 2013.p. 3–6 poster#765.

[66] Overton ET, Kitch D, Benson CA, Hunt PW, Stein JH, Smurzynski M (2013). Effect of statin therapy in reducing the risk of serious non-AIDS-defining events and nonaccidental death. Clin Inf Dis.

[67] Gili S, Grosso Marra W, D’Ascenzo F, Lonni E, Calcagno A, Cannillo M, Ballocca F, Cerrato E, Pianelli M, Barbero U, Mancone M, Di Nicolantonio JJ, Lavie CJ, Omedè P, Montefusco A, Bonora S, Gasparini M, Biondi-Zoccai G, Moretti C, Gaita F. Comparative safety and efficacy of statins for primary prevention in human immunodeficiency virus-positive patients: a systematic review and meta-analysis. Eur Heart J. 2016. pii: ehv734. 10.1093/eurheartj/ehv734 26851703

[68] Longenecker CT, Eckard AR, McComsey GA (2016). Statins to improve cardiovascular outcomes in treated HIV infection. Curr Opin Infect Dis..

[69] Weijma RG, Vos ER, Ten Oever J, Van Schilfgaarde M, Dijksman LM, Van Der Ven A, Van Den Berk GE, Brinkman K, Frissen JP, Leyte A, Schouten IW, Netea MG, Blok WL (2015). The effect of rosuvastatin on markers of immune activation in treatment-naïve human immunodeficiency virus-patients. Open Forum Infect Dis.

[70] Hileman CO, Turner R, Funderburg NT, Semba RD, McComsey GA (2016). Changes in oxidized lipids drive the improvement in monocyte activation and vascular disease after statin therapy in HIV. AIDS.

[71] Nou E, Lu MT, Looby SE, Fitch KV, Kim EA, Lee H, Hoffmann U, Grinspoon SK, Lo J (2016). Serum oxidized low-density lipoprotein decreases in response to statin therapy and relates independently to reductions in coronary plaque in patients with HIV. AIDS.

[72] Zidar DA, Juchnowski S, Ferrari B, Clagett B, PilchCooper HA, Rose S, Rodriguez B, McComsey GA, Sieg SF, Mehta NN, Lederman MM, Funderburg NT (2015). Oxidized LDL levels are increased in HIV infection and may drive monocyte activation. J Acquir Immune Defic Syndr..

[73] Gilbert JM, Fitch KV, Grinspoon SK (2015). HIV-related cardiovascular disease, statins, and the REPRIEVE Trial. Top Antivir Med.

[74] Lichtenstein KA, Hart RL, Wood KC, Bozzette S, Buchacz K, Brooks JT (2015). HIV outpatient study investigators. Statin use is associated with incident diabetes mellitus among patients in the HIV outpatient study. J Acquir Immune DeficSyndr.

[75] Bastida C, Also MA, Pericas JM, Letang E, Tuset M, Miró JM (2014). Rhabdomyolysis and severe hepatotoxicity due to a drug-drug interaction between ritonavir and simvastatin. Could we use the most cost-effective statin in all human immunodeficiency virus-infected patients?. Enferm Infecc Microbiol Clin..

[76] Chanson N, Bossi P, Schneider L, Bourry E, Izzedine H (2008). Rhabdomyolysis afterezetimibe/simvastatin therapy in an HIV-infected patient. NDT Plus.

[77] de Kanter CT, Keuter M, van der Lee MJ, Koopmans PP, Burger DM (2011). Rhabdomyolysis in an HIV-infected patient with impaired renal function concomitantly treated with rosuvastatin and lopinavir/ritonavir. Antivir Ther.

[78] Chauvin B, Drouot S, Barrail-Tran A, Taburet AM (2013). Drug-drug interactions between HMG-CoA reductase inhibitors (statins) and antiviral protease inhibitors. Clin Pharmacokinet.

[79] Gervasoni C, Riva A, Rizzardini G, Clementi E, Galli M, Cattaneo D (2015). Potential association between rosuvastatin use and high atazanavir trough concentrations in ritonavir-treated HIV-infected patients. Antivir Ther..

[80] Dinoso JB, Kim SY, Wiegand AM, Palmer SE, Gange SJ, Cranmer L (2009). Treatment intensification does not reduce residual HIV-1 viremia in patients on highly active antiretroviral therapy. Proc Natl Acad Sci USA.

[81] Llibre JM, Buzón MJ, Massanella M, Esteve A, Dahl V, Puertas MC (2012). Treatment intensification with raltegravir in subjects with sustained HIV-1 viraemia suppression: a randomized 48-week study. Antivir Ther.

[82] Hummelen R, Vos AP, van’t Land B, van Norren K, Reid G (2010). Altered host-microbe interaction in HIV: a target for intervention with pro- and prebiotics. Int Rev Immunol.

[83] Stiksrud B, Nowak P, Nwosu FC, Kvale D, Thalme A, Sonnerborg A (2015). Reduced levels of D-dimer and changes in gut microbiota composition after probiotic intervention in HIV-infected individuals on stable ART. J Acquir Immune Defic Syndr.

[84] Villar-García J, Hernández JJ, Güerri-Fernández R, González A, Lerma E, Guelar A (2015). Effect of probiotics (Saccharomyces boulardii) on microbial translocation and inflammation in HIV-treated patients: a double-blind, randomized, placebo-controlled trial. J Acquir Immune Defic Syndr.

[85] González-Hernández LA, Jave-Suarez LF, Fafutis-Morris M, Montes-Salcedo KE, Valle-Gutierrez LG, Campos-Loza AE (2012). Synbiotic therapy decreases microbial translocation and inflammation and improves immunological status in HIV-infected patients: a double-blind randomized controlled pilot trial. Nutr J.

[86] d’Ettorre G, Ceccarelli G, Giustini N, Serafino S, Calantone N, De Girolamo G (2015). Probiotics reduce inflammation in antiretroviral treated, HIV-infected individuals: results of the “Probio-HIV” clinical trial. PLoS ONE.

[87] Longo V, Bonato M, Bossolasco S, et al. Brisk walking improves inflammatory markers in cART-treated patients. Abstract 763. 21st Conference on retroviruses and opportunistic infections (CROI). Boston; 2014.

[88] Ogalha C, Luz E, Sampaio E, Souza R, Zarife A, Neto MG (2011). A randomized, clinical trial to evaluate the impact of regular physical activity on the quality of life, body morphology and metabolic parameters of patients with AIDS in Salvador, Brazil. J Acquir Immune Defic Syndr.

[89] Fillipas S, Oldmeadow LB, Bailey MJ (2006). A six-month, supervised, aerobic and resistance exercise program improves self-efficacy in people with human immunodeficiency virus: a randomised controlled trial. Aust J Physiother..

[90] Home-based exercise for management of HIV-associated cardiovascular disease (NCT01377064). http://www.clinicaltrials.gov/ct2/show/NCT01377064

[91] Effectiveness of team intervention over 12 months in reducing modifiable CVD risk factors on Framingham 10yr risk scores outcomes in HIV-1 subjects on antiretroviral therapy (NCT01436136). http://www.clinicaltrials.gov/ct2/show/NCT01436136

[92] Atherosclerotic risk and response to exercise intervention in HIV+ children (NCT00908284). http://www.clinicaltrials.gov/ct2/show/NCT00908284

[93] Effects of an exercise program on metabolic parameters of patients with a HIV infection. (NCT00910936). http://www.clinicaltrials.gov/ct2/show/NCT00910936

[94] d’Ettorre G, Ceccarelli G, Giustini N, Mastroianni CM, Silvestri G, Vullo V (2014). Taming HIV-related inflammation with physical activity: a matter of timing. AIDS Res Hum Retrovir.

[95] Saumoy M, Alonso-Villaverde C, Navarro A, Olmo M, Vila R, Maria Ramon J, Yacovo SD, Ferrer E, Curto J, Vernet A, Vila A, Podzamczer D (2016). Randomized trial of a multidisciplinary lifestyle intervention in HIV-infected patients with moderate-high cardiovascular risk. Atherosclerosis.

[96] Tseng ZH, Secemsky EA, Dowdy D, Vittinghoff E, Moyers B, Wong JK, Havlir DV, Hsue PY (2012). Sudden cardiac death in patients with human immunodeficiency virus infection. J Am Coll Cardiol..

[97] Esser S, Gelbrich G, Brockmeyer N, Goehler A, Schadendorf D, Erbel R, Neumann T, Reinsch N (2013). Prevalence of cardiovascular diseases in HIV-infected outpatients: results from a prospective, multicenter cohort study. Clin Res Cardiol.

[98] Esser S, Eisele L, Schwarz B, Schulze C, Holzendorf V, Brockmeyer NH, Hower M, Kwirant F, Rudolph R, Neumann T, Reinsch N (2014). Rates of cardiovascular events and deaths are associated with advanced stages of HIV-infection: results of the HIV HEART study 7, 5 year follow-up. J Int AIDS Soc.

[99] Petoumenos K, Reiss P, Ryom L, Rickenbach M, Sabin CA, El-Sadr W, d’Arminio Monforte A, Phillips AN, De Wit S, Kirk O, Dabis F, Pradier C, Lundgren JD, Law MG, D:A:D study group (2014). Increased risk of cardiovascular disease (CVD) with age in HIV-positive men: a comparison of the D:A:D CVD risk equation and general population CVD risk equations. HIV Med.

[100] Carballo D, Delhumeau C, Carballo S, Bähler C, Radovanovic D, Hirschel B, Clerc O, Bernasconi E, Fasel D, Schmid P, Cusini A, Fehr J, Erne P, Keller PF, Ledergerber B, Calmy A, Swiss HIV Cohort Study and AMIS registry (2015). Increased mortality after a first myocardial infarction in human immunodeficiency virus-infected patients; a nested cohort study. AIDS Res Ther.

